# Retinal Microvascular Changes after Intravitreal Triamcinolone Acetonide in Diabetic Macular Edema

**DOI:** 10.3390/jcm12103475

**Published:** 2023-05-15

**Authors:** Fusae Kato, Miho Nozaki, Aki Kato, Tsutomu Yasukawa

**Affiliations:** 1 Department of Ophthalmology, Toyota Kosei Hospital, Toyota 470-0396, Japan; 2 Department of Ophthalmology and Visual Science, Nagoya City University Graduate School of Medical Sciences, Nagoya 467-8601, Japan; 3 Department of Ophthalmology, Nagoya City University East Medical Center, Nagoya 464-8547, Japan

**Keywords:** diabetes, diabetic macular edema, optical coherence tomography angiography, vessel density, intravitreal triamcinolone acetonide, microaneurysms, acetonide

## Abstract

Intravitreal injection of triamcinolone acetonide (TA) is essential for clinical treatment in patients who insufficiently respond to vascular endothelial factor inhibitors for diabetic macular edema (DME). The aim of this study was to investigate microvascular changes treated with TA using optical coherence tomography angiography (OCTA). After TA in twelve eyes of eleven patients with central retinal thickness (CRT), there was a 20% or more reduction observed. Visual acuity, the number of microaneurysms, vessel density, and the foveal avascular zone (FAZ) area were compared before and at 2 months after TA. At baseline, the number of microaneurysms was 2.1 ± 1.1 in the superficial capillary plexuses (SCP) and 2.0 ± 1.1 in the deep capillary plexuses (DCP), with a significant decrease post-treatment to 1.0 ± 1.0 for SCP and 0.8 ± 0.8 for DCP (SCP; *p* = 0.018, DCP; *p* = 0.008). There was significant enlargement of the FAZ area from 0.28 ± 0.11 mm^2^ to 0.32 ± 0.14 mm^2^ (*p* = 0.041). There was no significant difference in the visual acuity and vessel density of SCP and DCP. Results indicated that OCTA was useful for the evaluation of qualitative and morphological retinal microcirculation and that intravitreal TA may decrease microaneurysms.

## 1. Introduction

Diabetic macular edema (DME) is a major cause of visual loss in diabetic patients [1,2]. The first-line treatment for DME targets is anti-vascular endothelial factor (VEGF) therapy, while intravitreal injection of triamcinolone acetonide (TA) has been established as a second-line treatment in DME [3]. VEGF has been identified as being the most important factor in the pathogenesis of DME [4]. However, there are limitations associated with the current anti-VEGF therapy, as it only targets VEGF and not any of the other inflammatory molecules.

Although DME pathogenesis has yet to be fully clarified, hyperglycemia in diabetes activates a variety of biochemical pathways that lead to increased hypoxia, formation of reactive oxygen species (ROS), and inflammation that is associated with the production of cytokines and chemokines [5,6]. Vitreous fluid levels of VEGF, intercellular adhesion molecule (ICAM)-1, interleukin (IL)-6, monocyte chemotactic protein (MCP)-1, and pigment epithelium-derived factor (PEDF) were all elevated in severe DME [7]. These mediators have been shown to lead to endothelial junction breakdown and agglomeration of leukocytes. Furthermore, the leukocytes in diabetic retinopathy have been shown to be involved in capillary non-perfusion, endothelial cell damage, and vascular leakage in the retinal microcirculation [8,9,10].

Steroid therapy, which suppresses cytokine production in multiple ways, has remained an essential option for patients who are refractory to anti-VEGF treatment [11,12]. In Japan, although the intravitreal dexamethasone implant (Ozurdex®; Allergan, Inc., Irvine, CA, USA) has not been approved, a preservative-free formulation of triamcinolone acetonide (MaQaid® 40 mg/vial; Wakamoto Pharmaceutical, Tokyo, Japan) has been approved for intravitreal and sub-Tenon injections. En-face optical coherence tomography angiography (OCTA) is a novel technology that can be used to visualize the retina and choroid microcirculation without dye injection [13]. Intravitreal TA may be effective in persistent or frequent recurrent DME after anti-VEGF therapy. It is necessary to verify what changes are caused to the microcirculation after TA. The aim of this study was to evaluate qualitative and morphological retinal capillary changes in DME patients treated with intravitreal TA by using OCTA (XR Avanti® AngioVue; Optovue Inc., Fremont, CA, USA).

## 2. Materials and Methods

This retrospective study was conducted between May 2018 and October 2021 at the Nagoya City University Hospital. Institutional Review Board (IRB) approval (#60-10-0009) was obtained for the study protocol and procedures. The study adhered to the tenets of the Declaration of Helsinki.

All patients underwent a comprehensive ophthalmic evaluation that included spectral-domain OCT, OCTA, best-corrected visual acuity (BCVA), tonometry, slit lamp biomicroscopy, and indirect fundus examination. BCVA was determined using a Landolt C chart and converted from the decimal system to the logarithm of the minimum angle of resolution (logMAR). The obtained data were compared between the values at baseline before the TA injections and at 2 months after administration. All intravitreal injections of triamcinolone (MaQaid®, 4 mg/0.1 mL) were administered under topical anesthesia. Subsequently, we performed paracentesis of the anterior chamber.

The inclusion criteria were center-involved DME patients treated with intravitreal TA who had a central macular thickness (CMT) reduction of at least 20% after treatment and a CMT that was greater than 300 µm before TA. The exclusion criteria were (1) retinal photocoagulation within 3 months prior to enrollment, (2) history of vitrectomy surgery within 6 months, (3) macular ischemia, (4) poor OCTA image quality (scan quality index < 6/10), (5) refractive error > −6 diopters, (6) uncontrolled glaucoma, (7) uveitis, (8) vitreous hemorrhage, and (9) vitreomacular traction by fibrovascular proliferation in the macular area. A history of previous anti-VEGF injections within 3 months was not a criterion for exclusion.

A spectral-domain OCTA (AngioVue System, XR Avanti®) device was used in this study. This device employs a split-spectrum amplitude-decorrelation algorithm (SSADA) to reduce the effect of the bulk motion and improve the signal-to-noise ratio in the axial direction. The OCTA analysis divided the macular region into the whole image, fovea, and parafovea for each vascular network of the retina, in accordance with the Early Treatment Diabetic Retinopathy Study (ETDRS) classification for diabetic retinopathy. The software (AngioAnalytic) automatically calculated the vessel density in the different retinal vascular networks. This included the superficial capillary plexus (SCP) and the deep capillary plexus (DCP) in a 3 × 3 mm quadrant scan centered on the fovea. Automated OCT segmentation was performed using the AngioVue module. The SCP was segmented between internal limiting membrane (ILM) to outer boundary of the inner plexiform layer (IPL). The DCP was segmented between an inner boundary of the IPL and outer boundary of the outer PL (OPL). Vessel density was calculated as the proportion of the measured area occupied by blood vessels with flow, which was defined as pixels having decorrelation values above the threshold level.

**Figure 1:**
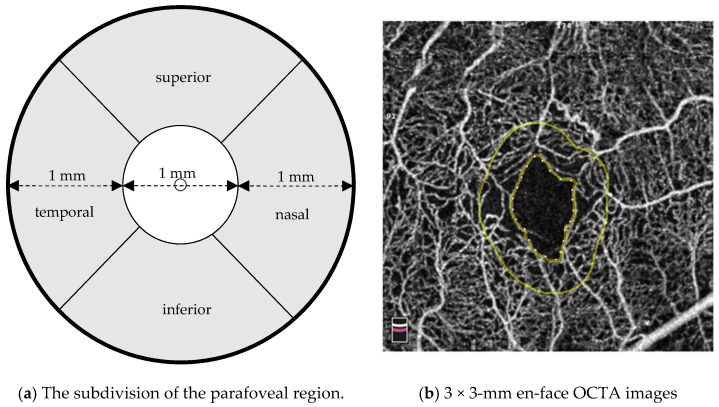
(**a**) Schematic diagram of the 3 mm Early Treatment Diabetic Retinopathy Study (ETDRS) grid centered over the fovea (right eye). The parafoveal region is the area (shown in gray) between the central 1 mm sector and the boundary of the 3 mm grid. The vessel density was calculated for each of the 4 sectors and the entire grid. (**b**) FD-300 is the vessel density of the whole inner retinal layer within a width of 300 μm around the FAZ region density. The area is shown between the inner and outer yellow perimeter.

Whole image vascular density was measured as the vascular density that was taken as a percentage within a circle that had a diameter of 3 mm from the center of the fovea. Parafoveal vascular density was measured as the vascular density that was taken as a percentage within the ring that was between 1 mm and 3 mm, and subsequently divided into four quadrants: temporal, superior, nasal, and inferior (Figure 1a). Scan protocols used the collected data to create the foveal avascular zone (FAZ) and the parafoveal capillary network. The quantitative analysis of FAZ was conducted using OCTA images of the whole inner retinal layer. FAZ was defined as the area encompassing the central fovea where there are no vessels. FD-300 is an OCTA-derived biomarker that measures the vessel density within 300 µm around the FAZ (Figure 1b). In fact, one of the advantages of using FD-300 as an OCTA biomarker is that segmentation of SCP/DCP is not required. As a result, this minimizes possible bias from segmentation error, which is commonly found in DME [14]. Acircularity index was defined as the ratio of the perimeter of the FAZ to the perimeter of a circle with equal area. A perfectly circular FAZ has an acircularity index equal to 1, with deviations from a circular shape leading to an increase in this metric. FAZs generally require axial length measurement to correct for individual retinal magnification, whereas an acircularity index does not [15]. FD-300, FAZ area, and acircularity index were all automatically measured using machine software. In contrast to that which is commonly done in many other papers, we did not attempt to differentiate between FAZ in the SCP and FAZ in the DCP, with our results simply referring to it as FAZ.

Microaneurysms in the SCP and DCP were separately counted. In line with other previous reports [16,17], we defined the microaneurysms as round, saccular, or fusiform capillary dilation. When counting the number of microaneurysms, which were obviously smaller in size after TA and considered to have been reduced, those that had no internal blood flow were not counted. Counting of the microaneurysms was performed twice in a masked fashion by one of the authors (FK). Results were obtained by analyzing the mean values of two measurements, after which intraclass correlation coefficients (ICCs) were also calculated. Repeatability was determined using the value of ICC.

Comparisons of the FAZ size, acircularity index, FD-300, the vascular density (SCP, DCP) and numbers of microaneurysms were performed at baseline (before TA) and at 2 months after administration.

All results are expressed as the mean ± SD. Data were collected and analyzed for paired values by the Wilcoxon signed-rank test. All statistical analyses were performed using the Statistical Package for Social Sciences (version 22.0; SPSS Inc., Chicago, IL, USA). Statistical significance was considered to be *p* < 0.05.

## 3. Results

A total of twelve eyes from eleven patients met the study inclusion criteria. Table 1 presents the baseline characteristics for all of the patients. The mean age of the patients was 65.9 ± 11.4 years (range: 44–81 years). Enrolled eyes had already undergone cataract surgery. Although one eye was a treatment-naïve case, the other eyes had been previously treated for DME (e.g., anti-VEGF therapy, sub-Tenon’s capsule injection of TA, intravitreal injection of TA, and/or navigated focal laser photocoagulation). There was significant CMT reduction from 452 ± 105 (316–637) μm to 274 ± 47 (197–394) μm after administration (*p* = 0.002)). There was no significant improvement in the mean logMAR BCVA (baseline logMAR BCVA was 0.27 ± 0.25, while at 2 months the logMAR BCVA was 0.23 ± 0.21, *p* = 0.144). Although there were no major adverse events, the IOP increased from 11 mmHg to 21 mmHg in one eye. In all cases, the increased IOP was managed using topical medication, with none of the patients requiring surgery.

### 3.1. OCTA Findings before and after Intravitreal Injection of Triamcinolone Acetonide

Table 2 shows the retinal microvasculature obtained by OCTA in a 3 mm × 3 mm area. After intravitreal TA, there was no significant difference observed in the vessel density in the observed SCP and DCP quadrants.

In the superior and the inferior area of the SCP, the vessel density was significantly increased (*p* = 0.028 in the superior area, *p* = 0.023 in the inferior area, respectively). There was enlargement of the FAZ area from 0.29 ± 0.10 (0.13–0.51) to 0.32 ± 0.13 (0.096–0.59) (*p* = 0.041). However, no significant differences were observed for the acircularity index and FD-300 between the values obtained before and after the TA. The ICC values were 0.70–0.78 (Table 3), and the collected data were considered to be reliable and useful for further analysis.

There was a significant reduction in the mean number of microaneurysms in the SCP and DCP. The baseline number of microaneurysms in the SCP area was 2.1 ± 1.1, while after the TA it was 1.0 ± 1.0 (*p* = 0.018). The baseline number of microaneurysms in the DCP was 2.0 ± 1.1, while after the TA it was 0.8 ± 0.8 (*p* = 0.008). A significant correlation was only found for CMT and for the number of microaneurysms in the DCP at baseline (R = 0.70, *p* = 0.016).

### 3.2. Representative Case

**Figure 2:**
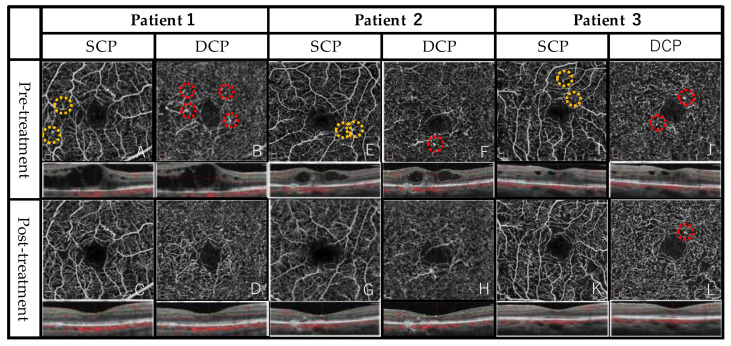
Representative images of the superficial capillary plexus (SCP) and the deep capillary plexus (DCP). The optical coherence tomography (OCT) scan through the fovea is shown directly below. Patient 1: optical coherence tomography angiography (OCTA) images were obtained from a 74-year-old woman. (**A**) Yellow circles indicate microaneurysms in the SCP before the intravitreal triamcinolone acetonide (TA). (**B**) Red circles indicate microaneurysms in the DCP before the TA. The central macular thickness (CMT) was 637 μm. The decimal BCVA was 0.5. (**C**,**D**) OCTA images are shown at 2 months after the intravitreal TA. The microaneurysms of the SCP and DCP disappeared. Macular edema had resolved and the CMT was 276 μm. The decimal BCVA was 0.7. Patient 2: OCTA images were obtained from an 81-year-old man. (**E**) Yellow circles indicate microaneurysms in the SCP before TA. (**F**) A red circle indicates microaneurysms in the DCP before TA. The central macular thickness (CMT) was 316 μm. The decimal BCVA was 0.4. (**G**,**H**) OCTA images are shown after the TA. The microaneurysms disappeared. Macular edema had resolved and the CMT was 197 μm. The decimal BCVA was 0.4. Patient 3: OCTA images were obtained from a 56-year-old woman. (**I**) Yellow circles indicate microaneurysms in the SCP before the TA. (**J**) Red circles indicate microaneurysms in the DCP before the TA. The CMT was 376 μm. The decimal BCVA was 1.2. (**K**) The microaneurysms of the SCP disappeared. (**L**) While 1 microaneurysm disappeared, the other remained (red circle). Macular edema had resolved and the CMT was 261 μm. The decimal BCVA was 1.2.

A 74-year-old woman who had DME for a few years had previously received an intravitreal injection of ranibizumab and TA. Five months prior to being enrolled in the present study, navigated laser photocoagulation was performed for the responsible microaneurysms at locations that were compatible with the thickest part of the retina. However, the DME proved to be refractory to these treatments. Prior to the intravitreal TA, the decimal BCVA was 0.5 and the CMT was 637 μm. OCTA images of the SCP and DCP were obtained (Figure 2—Patient 1). Prior to the TA, the FAZ area was 0.35 mm^2^, the acircularity index was 1.18, and FD-300 was 48.6%. After intravitreal TA, the decimal BCVA increased to 0.7 and CMT was reduced to 276 μm. After the TA, the FAZ area was 0.36 mm^2^, the acircularity index was 1.21, and FD-300 was 48.1%. The two microaneurysms in the SCP and four in the DCP observed before TA disappeared or became smaller after the administration of TA.

## 4. Discussion

In the present study, we used OCTA to document the changes in the retinal microcirculation of DME eyes treated with TA (Table 2). There were no significant changes in the vascular perfusion in most of the quadrants after the TA. However, there was significant enlargement of the FAZ area. The most peculiar finding of this study was the observed decrease in the number of microaneurysms in the SCP and DCP after the TA.

Past studies have investigated the repeated administration of anti-VEGF therapy on macular perfusion or microaneurysms in patients with DME. Many of these studies showed that repeated anti-VEGF therapy did not cause any treatment-related significant changes in the FAZ area or any capillary loss around the fovea [18,19,20,21]. The findings were not confounded in the patients on steroids. However, there are other studies that found that there were no significant changes in the vessel density (SCP and DCP) [22,23,24] and that there was a decrease in the FAZ area after an intravitreal dexamethasone implant [23,24]. In contrast, Carnota-Méndez et al. reported finding that there was a reduction in the vessel density and vascular perfusion in the absence of any changes in the FAZ area after the administration of dexamethasone [25]. Similar to some previous studies, we found in the present study that there was no significant change in the vessel density of the SCP and DCP, with the exception of two quadrants of the superior and inferior SCP. In contrast, the present study did show that there was significant enlargement of the FAZ area after the administration of TA (before: 0.29 ± 0.10 mm^2^, after: 0.32 ± 0.13 mm^2^ *p* < 0.041), which is not consistent with the previous report on the intravitreal dexamethasone implant [23,24,25]. To the best of our knowledge, at the present time, there have been no data reported on OCTA parameters that include information on microaneurysms after the intravitreal injection of TA.

Semeraro et al. evaluated a cohort of patients with retinal vein occlusion and reported finding a reduction in the arteriolar lumen diameter, as assessed by scanning laser Doppler flowmetry, after an intravitreal dexamethasone implant [26]. Wickremasinghe et al. used fundus photography to demonstrate that intravitreal triamcinolone reduced the caliber in both retinal arterioles and venules in eyes with refractory DME [27]. Dong et al. performed one of the largest histopathologic studies of microaneurysms that result from diabetic retinopathy [28]. Interestingly, they reported that the mean diameters of the microaneurysms with inflammatory cells (54.8 ± 29.9 μm) were significantly larger than those for the microaneurysms without inflammatory cells (37.2 ± 17.7 μm; *p* < 0.001). Furthermore, microaneurysms that contained inflammatory cells were more frequently located within regions that had capillary nonperfusion (34.4%), as compared with regions without capillary nonperfusion (15.5%) (*p* < 0.001). Based on these findings, the authors suggested that intraluminal aggregation of inflammatory cells may be a late feature of the microaneurysm lifecycle, as it increases in size. Thus, the upregulation of VEGF may play an important role in inflammatory cell recruitment and may precede the accumulation of inflammatory cells within microaneurysms. Intravitreal corticosteroids are known to block the production of inflammatory mediators and inhibit leukocyte aggregation [29]. Thus, the opening of capillaries and the subsequent increase in perfusion might lead to the noted changes in the microaneurysms. In the present study, microaneurysms disappeared or were downsized after the administration of TA. Thus, this might have been due to an improvement in nutrition and blood flow. There was also a significant increase in the vessel density in the superior and inferior quadrants of the SCP. In the areas that exhibited an increased vessel density, disconnected capillaries were observed, almost as if they were connected to a line. Although it is uncertain if such a small change might be clinically meaningful, this could potentially indicate that the capillary loss related to inflammation might be reversible.

In addition, the findings of our current study also showed that the TA treatment led to a significant increase in the FAZ area, even though there were no changes in the FAZ acircularity index or FD-300. Moreover, although there were no significant changes in the vessel density of the SCP and the DCP, there was an increase noted at some quadrants of the SCP. Thus, we speculate that the increased FAZ area was due to indirect effects, such as the displacement of capillaries and tissue perfusion in edema rather than vessel ischemia. It is difficult to provide a clear explanation for the cause of FAZ enlargement, but several possible reasons were considered, such as the following. It is possible that there was a reduction in the layer of capillaries or ganglion cell layer due to the anatomical effect associated with the reduction of the edema. A previous study reported that a larger FAZ was associated with a thinner CMT in healthy eyes [30]. Moreover, another study that evaluated glaucomatous eyes reported that the FAZ area had a significant negative correlation with the thickness of the macular ganglion cell and inner plexiform layer thickness [31]. These findings may imply that a thinner retina may have a lower metabolic requirement (low blood supply), which is associated with increases in the FAZ. Although the quality of the FAZ images was not definitively checked, it is likely that OCTA noise could have caused measurement errors due to macular edema. The swollen retinal parenchyma in the parafoveal area might press on the foveal tissue and decrease the FAZ. Moreover, it has also been reported that the presence of suspended scattering particles in motion (SSPiM) can lead to an overestimation of vessel density and vascular perfusion [32]. The SSPiM is frequently observed in vascular cystic macular edema, for which some of these cysts exhibit hyperreflective material when viewed by OCT. This material is composed of particles with a Brownian movement that gives a false-positive signal in OCT [33]. In addition, these types of cysts are potentially more sensitive to steroids than to anti-VEGF (similar to that of other types of lipid exudation as hard exudates or hyperreflective foci). As a result, a reduction or disappearance of these hyperreflective cysts after corticosteroid treatment would thereby result in a reduction of the OCTA flow signal.

The presence of microaneurysms is a biomarker that can be used to predict the response to anti-VEGF therapy and the severity of DME [34]. In several studies, the resolution of microaneurysms after anti-VEGF therapy has been reported [35,36]. However, in recent retrospective studies, numerous or large microaneurysms were reported to potentially be associated with a poor response to anti-VEGF therapy [37,38]. In our present study, we have shown that the number of microaneurysms may be decreased after the administration of intravitreal TA among cases with a history of anti-VEGF therapy or other DME treatment. Thus, intravitreal TA might be effective in patients with DME caused by leakage from microaneurysms.

Limitations of our present study include the fact that this was a retrospective study that only included a limited number of patients along with a short follow-up period. Furthermore, all eligible patients had a favorable response to the intravitreal injection of TA (CMT was reduced by at least 20%). It was not possible in the present study to obtain data on DME patients with poor/nonresponse to TA. In light of the low number of subjects with a poor response or nonresponse to TA, no statistical comparisons could be made between good and poor responses to TA. OCTA has significantly played a part in better characterizing vascular alterations in diabetes, and characteristics of detectable microaneurysms with this technology have been revealed [39]. When a blood flow rate in microaneurysms is below the threshold necessary to register as flow in the OCTA system, microaneurysms are less likely to be detected. In addition, because of retinal thickening that was located in the central area, OCTA images themselves, which are produced by an automated algorithm, might have contained several artifacts that ultimately lead to the misinterpretation of the images. Since these factors may have biased our results, further prospective studies with a larger number of patients and a longer follow-up period will need to be undertaken.

## 5. Conclusions

The improved DME after the administration of an intravitreal injection of TA showed there was a significant decrease in the number of microaneurysms. Thus, intravitreal TA might be effective in persistent or frequent recurrent DME after anti-VEGF therapy. Evaluation of macular perfusion by OCTA proved to be useful, and therefore, we believe that the analysis of microaneurysms may play an important therapeutic role in the management of DME. However, as there was an increased FAZ area observed after intravitreal TA, a more precise investigation will need to be undertaken in the future.

## Figures and Tables

**Table 1:** Patient characteristics.

Number of eyes/patients	12/11
Age (years) (mean ± SD; range)	65.9 ± 11.4 (44–81)
Sex (number of men/women)	4/7
Lens status	
phakia/pseudophakia	0/12
Eyes with diabetic retinopathy (%)	
Mild NPDR	1 (8.3)
Moderate NPDR	2 (16.6)
Severe NPDR	6 (50)
PDR	3 (25)
Types of DME morphology in eyes (%)	
cystoid macular edema	8 (66.7)
sponge-like diffuse retinal thickening	3 (25)
serous retinal detachment	1 (8.3)
Previous treatments in eyes (%)	
Anti-VEGF + TA + LP	3 (25)
Anti-VEGF + TA	3 (25)
Anti-VEGF + LP	1 (8.3)
TA + LP	1 (8.3)
Anti-VEGF	1 (8.3)
TA	1 (8.3)
LP	1 (8.3)
None (naïve case)	1 (8.3)

SD, standard deviation; NPDR, non-proliferative diabetic retinopathy; Anti-VEGF, vascular endothelial growth factor inhibitors; TA, sub-Tenon’s capsule and/or intravitreal injection of triamcinolone acetonide; LP, navigated focal laser photocoagulation.

**Table 2:** Optical coherence tomography angiography findings before and after intravitreal triamcinolone acetonide injection for diabetic macular edema.

		Pre-Treatment	Post-Treatment	*p* Value
LogMAR BCVA (mean ± SD)		0.27 ± 0.25	0.23 ± 0.21	0.144
CMT, μm (mean ± SD)		452 ± 105	273 ± 47	**0.002**
Number of microaneurysms				
SCP (mean ± SD)		2.1 ± 1.1	1.0 ± 1.0	**0.018**
DCP (mean ± SD)		2.0 ± 1.1	0.8 ± 0.8	**0.008**
Vessel density, %				
SCP (mean ± SD, range)	whole	40.4 ± 3.5	41.8 ± 3.1	0.100
	temporal	41.3 ± 4.8	42.1 ± 3.5	0.136
	superior	43.0 ± 5.4	45.8 ± 4.9	**0.028**
	nasal	41.7 ± 3.6	42.5 ± 4.5	0.722
	inferior	42.3 ± 4.8	44.7 ± 3.8	**0.023**
DCP (mean ± SD, range)	whole	40.7 ± 5.8	41.2 ± 2.3	0.424
	temporal	40.7 ± 5.9	41.8 ± 3.4	0.480
	superior	40.3 ± 6.1	42.4 ± 2.6	0.182
	nasal	41.3 ± 6.1	42.0 ± 3.6	0.209
	inferior	42.7 ± 7.2	42.6 ± 3.4	0.638
FAZ area, mm^2^ (mean ± SD, range)		0.29 ± 0.10 (0.31–0.51)	0.32 ± 0.13 (0.096–0.59)	**0.041**
FD-300, %		45.0 ± 3.6	44.8 ± 3.4	0.657
FAZ acircularity index		1.20 ± 0.07	1.19 ± 0.06	0.789

logMAR, logarithm of the minimum angle of resolution; BCVA, best-corrected visual acuity; CMT, central macular thickness; SCP, superficial capillary plexus; DCP, deep capillary plexus; FAZ, foveal avascular zone; FD-300, vessel density within a width of 300 μm around the FAZ; SD, standard deviation; bold values indicate *p* < 0.05.

**Table 3:** Reliability in the counting of microaneurysms. The ICC values were 0.70–0.78, and the collected data were considered to be reliable and useful for further analysis.

	Pre-Treatment		Post-Treatment	
Practice	P1	P2	P1	P2
Number of microaneurysms in the SCP (mean ± SD, range)	1.8 ± 1.2 (0–4)	2.5 ± 1.4 (0–4)	0.7 ± 1.0 (0–3)	1.2 ± 1.1 (0–3)
Mean	2.1 ± 1.1		1.0 ± 1.0	
ICC in the SCP	0.70		0.78	
Number of microaneurysms in the DCP (mean ± SD, range)	1.9 ± 1.3 (0–4)	2.0 ± 1.2 (0–4)	0.6 ± 0.7 (0–2)	1.0 ± 1.0 (0–3)
Mean	2.0 ± 1.1		0.8 ± 0.8	
ICC in the DCP	0.71		0.78	

P1, practice 1; P2, practice 2; SCP, superficial capillary plexus; DCP, deep capillary plexus; ICC, Intraclass correlation coefficients.

## Data Availability

The datasets generated during and/or analyzed during the current study are available from the corresponding author upon reasonable request.
